# Phytolith Formation in Plants: From Soil to Cell

**DOI:** 10.3390/plants8080249

**Published:** 2019-07-26

**Authors:** Muhammad Amjad Nawaz, Alexander Mikhailovich Zakharenko, Ivan Vladimirovich Zemchenko, Muhammad Sajjad Haider, Muhammad Amjad Ali, Muhammad Imtiaz, Gyuhwa Chung, Aristides Tsatsakis, Sangmi Sun, Kirill Sergeyevich Golokhvast

**Affiliations:** 1 Education and Scientific Center of Nanotechnology, Far Eastern Federal University, 690950 Vladivostok, Russia; 2 Department of Forestry, College of Agriculture, University of Sargodha, 40100 Sargodha, Pakistan; 3 Department of Plant Pathology, University of Agriculture, 38040 Faisalabad, Pakistan; 4 Center of Agricultural Biochemistry and Biotechnology (CABB), University of Agriculture, 38040 Faisalabad, Pakistan; 5 Soil and Environmental Biotechnology Division, National Institute for Biotechnology and Genetic Engineering, 38040 Faisalabad, Pakistan; 6 Department of Biotechnology, Chonnam National University, 59626 Yeosu-Si, Korea; 7 Department of Toxicology and Forensics, School of Medicine, University of Crete, Heraklion GR-71003, Crete, Greece; 8 Pacific Geographical Institute, FEB RAS, 7 Radio street, Vladivostok 690014, Russia

**Keywords:** biosilicification, biomineralization, siliplant1 (Sip1) protein, Lsi, phytoliths, palaeoenvironment reconstruction, silicon

## Abstract

Silica is deposited extra- and intracellularly in plants in solid form, as phytoliths. Phytoliths have emerged as accepted taxonomic tools and proxies for reconstructing ancient flora, agricultural economies, environment, and climate. The discovery of silicon transporter genes has aided in the understanding of the mechanism of silicon transport and deposition within the plant body and reconstructing plant phylogeny that is based on the ability of plants to accumulate silica. However, a precise understanding of the process of silica deposition and the formation of phytoliths is still an enigma and the information regarding the proteins that are involved in plant biosilicification is still scarce. With the observation of various shapes and morphologies of phytoliths, it is essential to understand which factors control this mechanism. During the last two decades, significant research has been done in this regard and silicon research has expanded as an Earth-life science superdiscipline. We review and integrate the recent knowledge and concepts on the uptake and transport of silica and its deposition as phytoliths in plants. We also discuss how different factors define the shape, size, and chemistry of the phytoliths and how biosilicification evolved in plants. The role of channel-type and efflux silicon transporters, proline-rich proteins, and siliplant1 protein in transport and deposition of silica is presented. The role of phytoliths against biotic and abiotic stress, as mechanical barriers, and their use as taxonomic tools and proxies, is highlighted.

## 1. Introduction

Phytoliths are microscopic amorphous silica structures that are produced within and between plant cells by precipitation and polymerization of silica, and this process is called biosilicification [1]. Biosilicification is widespread in the plant kingdom and it has attracted increasing attention in recent years [2]. Silicon (Si) is absorbed by roots as soluble silica (Si(OH)_4_) from the groundwater and it is carried to different parts of the plant system through the vascular system. The precipitation of silica inside the plant body takes place at neutral pH under ambient temperature and pressure conditions, and it largely depends on evaporation and metabolism of water, as it could be either “biologically induced” or “biologically controlled” [3]. Intracellularly, silica is deposited in both the cytoplasm and vacuoles of the plant cells whereas intercellularly phytoliths are deposited in almost all plant parts i.e., roots, stems, leaves, fruits, inflorescence, etc. In addition to silica, some of the plants also produce nonsilicaceous phytoliths that are composed of calcium oxalate (CaOX), calcium carbonate, calcium sulphate, calcium phosphate, magnesium oxalate, stronium oxalate, and stronium and barium sulfate. However, this is not the aspect that we will focus on in this review, and is described elsewhere [3].

Phytoliths are very resilient and they are often preserved as fossil records, because they survive death and decomposition of the plant; their inorganic nature helps them to resist the destructive forces and survive as sediments in diverse environments of land and ocean [1]. Their durability in soil and dry environments and distinctive shapes makes them increasingly accepted as new vegetation, environment, and climate proxies. Phytoliths record unique characteristics of past vegetation and environmental conditions which help to date a sample [4]. Phytoliths provide pieces of evidence for the distribution of taxa or vegetation and help in drawing more reliable inferences regarding palaeovegetation and in reconstructing the palaeoenvironments [5]. They can further be used to study their effect on other organisms i.e., grazers, ecosystems, biogeochemical cycles, and changes in the geosphere. This wide range of utility has resulted in the elucidation of Cretaceous-Cenozoic grass evolution and it has provided answers to many paleoecological questions regarding the co-evolution of plants and animals, diets of extinct animals, and vegetation change over the course of evolution, such as the spread of grasslands [6]. Phytolith analysis is considered as cross-disciplinary research owing to the diversity of disciplines and researchers involved, and it is emerging as an Earth-life superdiscipline [2]. However, this cross-disciplinary research has previously seen little standardization, but thanks to the efforts of the International Working Group on Phytolith Nomenclature who led the development of the first International Code for Phytolith Nomenclature 1.0 [7]. Another improvement in this area has been the development of an online database PhytCore ODB, which provides comparative keys for facilitating identification in plants [8]. 

Silicon is the second most abundant element on earth, comes to the researcher’s mind with the thinking of the age of research ‘*in silico*’ and the global rise of silicon valleys. Until now, we should have been able to understand the functions of biosilicification in plants, but it still remains an enigma when we think about the pathways and processes that are involved in phytolith formation and phytoliths’ role in plants [9,10]. The phytolith research is often ignored by mainstream literature regarding the plant functional diversity [2], which might be due to the fact that Si is not an essential element for plant metabolism [9,11]. However, understanding the mechanisms of Si deposition in plants and its nutritional role and phytolith contents now gains an increasing recognition as a plant functional trait as during the last two decades much progress has been made towards understanding the transport and nutritional role of Si in the plants [12,13]. 

This review focuses on the integration of recent knowledge and concepts that have been used to understand Si uptake, transport, and phytolith formation in plants. Here, we discuss the evolution of biosilfication in plants with an insight on proteins of biosilicification, how they interact in a coordinated manner to make phytoliths, and how the size, shape, and chemistry of phytoliths differ. Finally, we talk about the functional uses of phytoliths in different yet related fields.

## 2. Phytolith Formation in Plants

Higher plants, other photosynthetic organisms, such as diatoms and other eukaryotic lineages i.e., siliceous sponges, radiolarians, etc., play an important role in global Si cycling, and it has been suggested that biosilicification has driven variation in the global Si cycle over the geological time [14]. Vegetation, particularly tree species and grasses, strongly impacts the terrestrial silicon cycle by absorption and restitution via litterfall or plant death [15]. Thus, the dynamics of Si in terrestrial ecosystems is largely governed by pedogenesis and its relationship with the plant community and its diversity [16]. Hence, the terrestrial biogeochemical Si cycle drives the global Si cycle, which further stresses the importance of silica deposited within living tissues of plants and it highlights the importance of considering Si, not just as an element residing within plant tissue but to a geological and biogeochemical scale [14,17]. While the global Si cycle is well understood at the interface of earth and life sciences [ see “the global silicon cycle” in 9] a clearer picture of silica transport and deposition as phytoliths in plants is presented here. Silicon enters the plant in the form of silicic acid, along with the water, and it is carried away towards various plant parts by transpiration stream through the vascular system. Upon transpiration, silicic acid gets concentrated in plant tissues as solid hydrated silica and it is precipitated as phytoliths. In the water-limited conditions these phytoliths get resolubilized and end up outside of the plant bodies and join the flux of silicic acid into the oceans [9,18]. This conversion is not as simple, as uptake, transport, deposition, resolubilization, and ending up in the ocean is a complex mechanism that involves various known and unknown pathways. Additionally, it is worth noting that biosilicification by living organisms, including plants, results in colossal amounts as compared to the industrial processes (gigatons vs megatons, respectively) [19]. A clear understanding of the bioavailability of phytolith and the mechanism behind their formation is crucial for their applications in paleobotanic and archaeological record interpretations, as well as in the improvement of crop plants. 

### 2.1. Silicon Uptake and Transport

The Si uptake potential of hundreds of plants (>500 plants) has been reported making way towards classifying plants as Si accumulators (rice (*Oryza sativa*), horsetail (*Equistium arvense*), sugarcane (*Saccharum officinarum*), sorghum (*Sorghum bicolor*), and other grasses), and Si nano-accumulators (most of the dicots) since the discovery of phytoliths and presence of Si in plants. The variation in Si concentration in plant tissue on dry weight basis leads us towards a better understanding of Si uptake [20]. Most soil contains soluble silica as monosilicic acid [Si(OH)_4_] at pH 9 at a variable concentration of 0.01 to 2.0 mM, depending on the soil type [21]. The Si uptake by plants depends on the conversion of Si in soil into plant-available silicic acid, as was observed in the case of wheat grown on different pools of Si i.e., quartz sand (crystalline), anorthite powder (crystalline), and silica gel (amorphous). While silica-gel treatment showed significantly higher Si-uptake rates, in the other two cases, it was not different from control [22]. The kinetic studies have demonstrated that Si concentration in root symplastic solution increases in higher external Si concentrations [23]. Silicic acid is radially taken up through root cortical-cells either through diffusion or in an energy-dependent manner [24,25]. A channel-type Si transporter gene Lsi1 (low silicon 1) transports Si across the plasma membrane from apoplast to a plant cell [26,27]. The passive transport has been reported in rice, barley (*Hordium vulgare*), wheat (*Triticum aestivum*), maize (*Zea mays*), cucumber (*Cucumus sativus*), pumpkin (*Cucurbita moschata*), and soybean (*Glycine max*) [12]. Within roots (as observed in case of rice) Si arrives at the distal side of exodermal cells into the symplast by Lsi1 and then exported to proximal side apoplastic connections by an efflux transporter Lsi2 (low silicon 2). Silicon is further imported into the symplast of the endodermis by Lsi1 that was localized on the distal side of the endodermis and exported to the stele by Lsi2 that was localized on the proximal side of the endodermis [26,28]. While this Si transport mechanism is only specific to rice, the other plants do depend on both transporter genes, but contrasting to Lsi1 and Lsi2 in rice, in barley and maize these two genes are located on different cell layers i.e., epidermal, hypodermal, and cortical cells, from which Si is transported to endodermis [26,27,28]. This difference in Si transport in rice and other monocot plants might be due to differences in their root structure and is discussed in detail elsewhere [12], and has been previously described as the ability of the roots [29]. An interesting consideration in variable transport mechanisms among Si accumulators could be polar versus non-polar localization of silicon transporters, however the polar localization has only been seen in rice, owing to Casparian strips in exodermis and aerenchyma formation [30,31]. Table 1 summarizes the different expression patterns and localization of silicon transporters from various species. Silicon transporters show variable patterns of expression in different species in response to Si application [32]. In rice (*OsLsi1* and *OsLsi6*) and soybean (*GmNIP2-1* and *GmNIP2-2*), the silicon transporters are downregulated, but upregulated in cucumber while in maize, barley, and wheat the expression of Lsi1 is unaffected [26,33,34]. These observations suggest that the mechanism regulating the expression of channel-type silicon transporters differs in different species and calls for further investigations involving the promoter regions of these genes. Subcellular mislocalization also contributes to an inability to transport Si, as observed in cucumber, where an influx transporter *CmLsi1* was localized in the endoplasmic reticulum [35]. The allelic variation within silicon transporter sequence might affect subcellular localization, as well as Si transport into the roots, which is further discussed in Section 3 [36]. Apart from Lsi1 and Lsi2, the Lsi1 homologs are also sometimes involved in Si transport in roots. In barley, *HvLsi6* has been found to be involved in Si uptake in roots where it was localized in the plasma membrane [37]. Taken together it could be stated that the energy-dependent uptake of Si in roots is under harmonized control of both channel-type and efflux transporters. After reaching the roots, Si is then carried to the shoot via transpiration flow through vascular bundles. However, some studies have suggested that it is actually the transpiration from the shoot that triggers diurnal changes in root NIP’s expression [38]. In xylem, Si is present as monosilicic acid and disilicic acid (in 7:1 ratio as observed in wheat plants) [39]. Silicon is then transported out of xylem (xylem unloading) and it is distributed in the leaf [34] and possibly in the panicles. It was previously suggested that xylem loading of Si in rice was mediated by a silicon transporter gene (Lsi6), but by passive diffusion in cucumber suggested that different species have evolved differently [40]. It has been shown in many species such as rice and barley that Lsi6 (by *OsLsi6* in rice and *HvLsi6* in barley) is involved in xylem unloading in the shoot (intervascular transfer in nodes), leaf blade, and sheath. The coupling of *HvLsi6* and *HvLsi2* has been suggested to control the intervascular Si transfer in nodes, where “Si translocated via the enlarged vascular bundles is unloaded to the transfer cells by *HvLsi6*, followed by *HvLsi2* to reload Si to the diffuse vascular bundles, which are connected to the upper part of the plant, especially the panicles, the ultimate Si sink” [37]. Another homolog of Lsi2 i.e., *OsLsi3,* has also been reported in nodes that are located in the parenchyma tissues between enlarged vascular bundles and diffuse vascular bundles. The discovery of this third gene suggested that orchestration of Lsi2, Lsi3, and Lsi6 perform the intervascular Si transfer, which is required for preferential distribution of Si [41]. As discussed in the introduction section that phytoliths are found in all plant parts including inflorescence and fruits, it could be speculated that some form of Lsi6 or other silicon transporters may control this transport (see Figure 4 in [12]). During the maturation of plants, silicic acid is selectively transported to the panicles (in case of rice), where it is deposited in the inflorescence brackets. This transport could be due to the increasing sink strength of panicles [41,42], while the mechanism of involvement of silicon transporters still remains unexplored. A study on silicon transporters in cucumber has reported the expression of Lsi1 homologs *CsiT-1* and *CsiT-2* in petals, which suggests the involvement of silicon transporter in Si transport to reproductive parts of the plants [43]. It is to be noted here that the expression of the Lsi1 homologs in other Gramineae members is confined to roots only [44]. Contrary to this, when a wheat silicon transporter i.e., *TaLsi1* was expressed in Arabidopsis, no expression could be observed in flowers hinting towards the notion that monocots and dicots might have different strategies to transport Si to flowers, but this has to be explored yet [45]. The expression of *ZmLsi1* and *ZmLsi6* in maize kernel has already been reported which indicates the involvement of channel-type transporters in Si transport to immature and mature kernels [46]. The Si transport dynamics is well understood, and it is now known that rice has a system that maximizes the investment efficiency of Si uptake by manipulating the expression of silicon transporters under diurnal variation [47]. The presence of only 1 Lsi1 homolog and four Lsi2 homologs has been reported in rice, but the functional characterization of a large number of homologs in dicot species is still limited, and a complete understanding of the mechanism of Si transport from stem to leaves and inflorescence is still missing. 

### 2.2. Si Deposition and the Formation of Phytoliths

**Figure 1:**
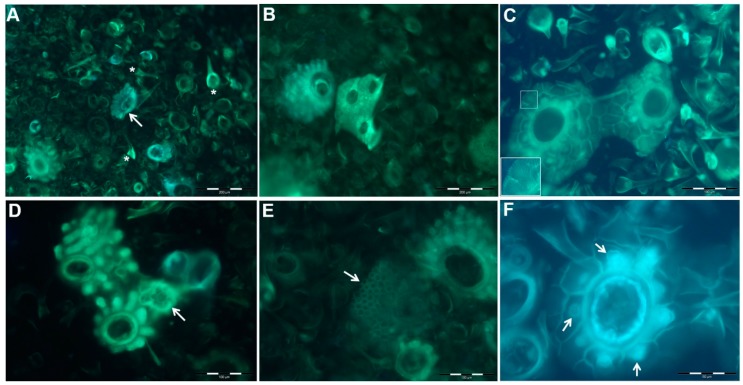
Biogenic silica deposition in hemp leaves after acid extraction and fluorescence imaging with (2-(4-pyridyl)-5-((4-(2-dimethylaminoethylaminocarbamoyl)methoxy)phenyl)oxazole) (PDMPO). (**A**) Isolated trichomes, some of which with basal cells; (**B**) Isolated trichomes and two adjacent trichomes with basal and epidermal cells; (**C**) Trichomes with epidermal cells showing replicas of waxy “wrinkles” (boxed region and bottom magnified image thereof); (**D**) Compound trichome base (arrow) interspersed with single-celled trichome bases; (**E**) Reticulated structure possibly representing a developmental stage of trichomes with compound bases; and,(**F**) strongly labelled structures inside epidermal cells possibly representing vacuoles/large vesicles. The scale bars in panels A–C are 200μm, in D–E 100μm and in F 50μm (with permissions from [57]).

Once in the plant body, silicic acid is deposited as phytoliths in plant in conjunction with water loss through transpiration in almost all levels i.e., organelle (cell wall, lumen), cell (guard cells of stomata), tissue, and organ (roots, shoots, leaves, and inflorescences) [1,26] (Figure 1). In terms of chemical changes from absorption to deposition, silica is absorbed in the form of orthosilicic acid in monomeric form from the soil into plant roots. This molecule is weakly acidic and it is composed of Si tetrahedrally-coordinated to four hydroxyl groups. Once in the body, owing to transpiration, its concentration increases to 100–200 mg/Kg [48]. The deposition than form dimer anions (Si(OH)_3_OSi(OH)_2_O- and it continues to form dimers, trimers, oligomers, and polymers with different Si-O-Si bond angles and distances and it is eventually precipitated as silica [49]. It is here that the silica is either deposited in the forms of sheets, globular or fibrillar form. Currie and Perry [19], in their earlier studies, discussed that the thickness of the deposited silica actually depends upon the location within the plant [48,50,51,52], and concluded that biogenic silica particles show a narrow particle size distribution for specific structural motifs. However, regarding size, a different opinion also exists [10], which is elaborated in detail in Section 4. Plant silicification occurs in cell lumens, intercellular spaces, and cell-walls under the control of different silicification mechanisms, which is discussed below, respectively. 

Biosilicification is controlled by cell development and tissue maturation and it is believed that during the early developmental phases of juvenile organs there is little or no Si deposition, with the exception of specialized silica cells [53,54]. In diatoms, the deposition and polymerization of silica are now well characterized, and it has been established that different cell-wall proteins i.e., frustulins, pleuralins, silaffins, and p150 family members work in a coordinated and controlled manner to form silicified cell walls [55,56]. 

Parry and Smithson [53] stated that silica deposition in plants is controlled by cell development and it depends on the tissue maturation, but our understanding about this process in vascular plants is poor [58]. This process, in comparison to diatoms, is much less under cellular control [4]. The process of silica polymerization is completed in a stepwise manner in oat (*Avena sativa* L), where the silicic acid is irreversibly formed to amorphous silica (SiO_2*n_H_2_O). The silica is deposited as a non-crystalline (13.5% bound water in silica) structure i.e., silica gel, which then grows into amorphous solid structures [59]. During the development, a sequence of long and short cells arise in the interphase of intercalary meristem cells of an internode; the short cells have the ability to develop into a pair of cork and silica cells, stomata cells, or trichomes. For silicification, (1) a pair of morphologically similar cells (silica and cork cell) appear and (2) then differentiate in size and shape. In ryegrass (*Lolium temulentum*), upon differentiation, silica-cells show rapid changes, such as enlargement of vacuoles and increase in number of rough endoplasmic reticulums and mitochondria. The Golgi bodies and other small vesicles appear. (3) The nucleus of the silica cell perishes. (4) Upon degradation of the nucleus, fibrillar elements arise from this and other cytoplasmic elements and then spread through the cell lumen followed by the formation of thickened cell walls around the cork and silica cell [60]. This typical process suggests that silica deposition or per se phytolith formation is a metabolically controlled mechanism. In this case, the minerals that formed in silicified cells are restricted between the cell wall and cell membrane (paramural) [61]. This process of silification has also been studied in sorghum and it was suggested that transpiration is not exclusively accountable for silification, as only young leaves were able to accumulate silica in silica cells and there might be a biological process that is involved for the local silica polymerization [62,63]. Furthermore, if the silicification is too dependent on transpiration, the guard cells of the stomata should be heavily silicified and all of the transpiring leaves should continue accumulating silica in their silica cells while the process of silicification should continue even after the leaf matures [64]. Interestingly, this never happens as the guard-cell silicification is a slow-paced process and advances with age, which implies that mineralization is not a uniform process across all cell types [65]. When considering the fact that silicification follows the tissue maturation, it is important to relate it with the developing leaf tissues and cells, particularly the silica cells. In a developed-leaf, there exists a gradient of tissue maturation, which should also represent a gradient of silicification. The silicic acid arrives in silica cells, by the process that is supported by transpiration stream and silicon transporters, from environments of low concentration (<2 mM) and is deposited intercellularly as silica [49,66]. The silicification process in these cells is initiated at the cell-periphery and not at the position of the vacuoles [67]. This process continues as long as the cells are viable [68]. This silica biogenesis in silica cell was recently elucidated by Kumar, et al. [69] in sorghum to be under the molecular control of a siliplant1 protein (SIp1). The previous report by the same group on sorghum confirmed this, where they discovered that the silica cells are still viable in non-silicified conditions and may die in such conditions strongly suggesting that “silica cells lose their nucleus and cytoplasm, become empty and subsequently get silicified in a passive way” [62]. The SIp1 protein is transcribed, translated, and then post-transcriptionally modified during the time when the cell prepares itself for silicification. The SIp1 protein remains packed inside the vesicles and is stored in the cytoplasm until the cells reach a certain condition, where silicification is initiated. Upon release, the peptide contacts the mineral and coordinates the silicification. This spatiotemporal release of SIp1 needs to be further explored to determine what signals the vesicles containing SIp1 to fuse with the membrane and release it. It is evident from the work on sorghum that silicification in silica cells is dependent on the SIp1 protein, while silicification in the cell wall and other cells do not involve this protein [69]. This raises a question, whether there are possibilities that this protein is not functional in other cell types because it might not receive the signals prior to or during the programmed silica cell death? The presence of this protein in immature inflorescence could possibly give an answer to the question of what types of signals are required for its release. 

While silicification of silica cells is under the control of tissue maturation and SIp1 protein, the lumen of other cell types still silicify, such as bulliform cells, prickle hairs, fusoid cells, and leaf micro-hairs in leaves [64]. In inflorescence, the different cell-types, such as prickle hairs, papillae, long cells, epidermal cells, and macro-hairs silicify in the spikelet (within glume and lemma) [70,71,72]. First, it is very clear that the deposition process of silica differs, depending on the cell type [73,74]. Second, each cell type in different tissues accumulates a variable amount of silica, which in some cell types continues after leaf maturation e.g., bulliform cells, micro-hairs, and prickle hairs in the aging leaf tissues of bamboo grass (*Sasa veitchii*). Contrarily, the silica cells in this species are only silicified during leaf development and not after the maturation [73]. It is important to know that in grasses, e.g., bamboo, the basal cells of micro-hairs are silicified before their exposure to the outer environment suggesting that factors other than transpiration are also involved [75]. The research on sorghum has described that the silification process is initiated at a cell-wall, followed by the passive filling of the lumen post-death of the cells. Does this mean that the base cells of leaf micro-hairs are silicified in the same patterns as the silica-cells? Third, the age-dependent increase of micro-hairs suggests transpiration dependency. Finally, it remains unknown whether there are any types of proteins that are involved in the silicification process of cells other than silica-cells in leaves. 

The cells in inflorescence also express theSIp1 protein. While it is not known whether the protein is localized specifically in glume or lemma, there remains a question of what types of post-transcriptional modifications might be mediating silica deposition? It seems that the outer tangential cell-wall of papillae and prickle hair tips are already silicified at the stage of emergence similar to the basal cells of micro-hairs in bamboo leaves. As far as lumen is concerned, Kumar, Soukup and Elbaum [62] proposed that in dead cells this process might be completed in two steps i.e., at the tips and the cell wall induced by wall materials, followed by spontaneous precipitation inside the lumen triggered by degrading cell death and evapo-transpiration. We need to consider the conditions cells are going through during the cell death to understand this completely. It is important to know whether the silicification starts prior to cell death in “active cells” of not. Further explorations on how this silica precipitation in the lumen is triggered by protoplast degradation could lead to a better understanding of the transcriptional and post-transcriptional events, leading to the continued silicification after the cell death. The fact that these cell types have stomata suggests some role of transpiration. However, the cells devoid of stomata also silicify. Following the evens of silicification of macro-hairs in the outer epidermis of the lemma of canary grass (*Phalaris canariensis*), it was proposed that cell-wall templates the silica [50,51,71,76]. 

Kumar, Soukup and Elbaum [62] reviewed evidences regarding different modes of silification in greases and concluded that the cell wall silification can either be spontaneous or direct, except for paramural silification. Mature and intensively transpiring organs have continuous Si supply that is aided by transpiration stream, which is deposited on non-silicified cell wall passively without being metabolically controlled by the host cells upon dehydration. The controlled cell-wall silification remains an interesting matter of discussion until now [4,51,76]. The silica on the cell-wall is directly laid down onto the carbohydrate material. The interaction of cell wall polymers and Si has been widely explored in rice and horsetail. However, it is to be noted that the plant cell walls vary considerably in their structure, composition, and architecture between different types of cells and plant species [77]. The cell wall is composed of various polymers i.e., cellulose, cross-linking glycans (xyloglucan, glucuronoarabinoxylan, and mannans), pectin (homogalacturonans and rhamnogalacturonanas), lignin (cross-linked coumaryl, coniferyl, and sinapyl alcohols), proteins, and glycoproteins (enzymes, hydroxyproline-rich proteins) [77,78]. A positive correlation between Si content and cell-wall polymers is already established, and it is known that these polymers favor precipitation of Si aggregates. The structure of silica deposited on the cell wall is dependent on newly laid-down polysaccharide, as in undersaturated silicic acid conditions, callose [β-(1→3)-D-polyglucose] induces silica deposition [79], and it is possible that, similarly, other polysaccharides might play such role in cell wall silicification. For example, the silicification of organs with little or no transpiration i.e., in the outer epidermal cells and macro hairs in lemma the silicification proceeds with the wall thickening. Additionally, the capacity to store or bind with silica differs among cell wall polymers in the order of lignin>polysaccharides>lipids during lignocellulose fractionation of rice straw [80]. The unique cell walls of *Poales* (e.g., rice) contain a high level of (1;3,1;4)-β-D-glucan (mixed-linkage glucan (MLG)) and share similarities with that of horsetail, even though both species are evolutionarily distinct. Another similarity between both species is the high Si accumulation, which suggests a functional correlation with MLG [81,82]. A recent study by Kido, et al. [83] stated that “there is at least a temporal (indirect or direct) link between MLG and Si during and after the leaf expansion process”, and that the Si distribution within rice leaf blade tissues requires MLG. However, the study also noted that MLG was rapidly degraded in primary cell walls, rendering the notion that it is very unlikely for silica to remain bound to MLG. While this interaction remains less understood, other studies on the cell wall and Si interaction have found a positive correlation between Si and polymer feature i.e., cellulose crystallinity, arabinose substitution degree of xylans and sinapul alcohol proportion of lignin [84]. The exact role and the process of deposition of Si with cell wall polymers remains poorly understood, however it is now established that composition of monosaccharides that is associated with pectins and hemicelluloses is altered in Lsi1 stiff brome (*Brachypodium distachyon*) mutant (Bdlsi1-1) and there is rearrangement in the degree and pattern of homogalacturonan methylesterification [85]. The exact pattern of silica deposition on the cell wall matrix is still to be elucidated, while the form of the non-phytolith Si that is deposited on the exterior of the rice straw is Si(OR)_x_, Si-O-Si, SiO_x_, and Si-C, respectively [86]. It is suggested that non-phytolith Si is deposited on cellulose micro-fibrils through hydrogen bonding or condensation reaction with cellulose. This has been observed in the formation of bacterial cellulose/silica hybrid that was fabricated by mimicking biocomposites [87]. Hodson [4] suggested that the molecular structures of templates define the propensities of silicification of particular structures that are able to heavily silicify themselves. This process involves modeling, dissolution, and remodeling of the silica structures. 

## 3. Proteins/Genes of Biosilicification in Plants

**Figure 2:**
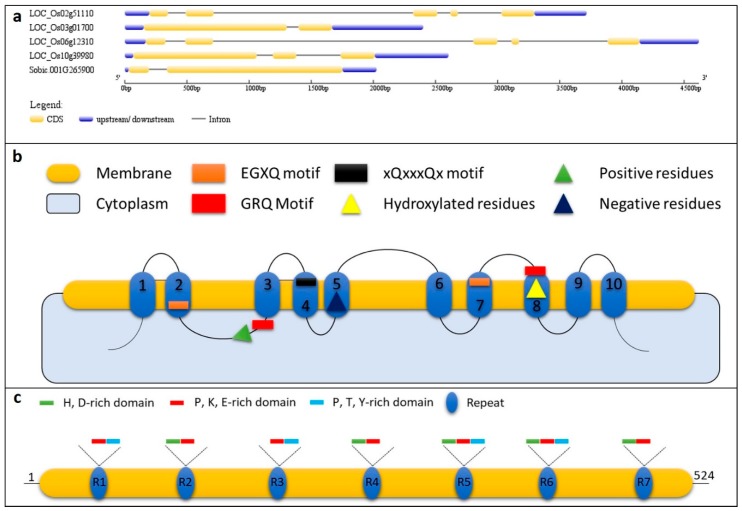
(**a**) Gene structure of four rice low-silicon genes Lsi1, Lsi2, Lsi3, and Lsi6 and a sorghum SIp1 gene. Gene sequences were retrieved from Phytozome and visualized in Gene structure display server. (**b**) A generalized structure of silicon transporter gene (modified from [100]) and (**c**) sorghum SIp1 gene (modified from [69]).

The deposition of Si in the form of phytoliths is fascinating in many ways. When visually observed, they are seen as exquisitely beautiful and intricate structures. Interestingly, the process of phytolith formation occurs under physiological conditions with temperatures that range from 0 to 40 °C, neutral pH, and ambient pressure. The secret behind this marvellous process of sculpturing silica at nanoscale must be the genes and proteins that are involved in the transport and deposition of Si. So far it is known that silicon transporters, silaffins, silicateins, silacidins, and silicases play essential roles in the process of biosilfication in different living organisms i.e., diatoms, sponges, and plants [10,55,88,89]. However, specifically in plants, most of the phytolith research is dedicated to the discovery of silicon transporters and Nod26-like major intrinsic proteins (NIPs). The search for silicon transporters started when this gene family was identified in the marine diatom (*Cylindrotheca fusiformis*) [90]. To understand the mechanism of Si transport in plants, one of the silicon transporter genes from diatom was transformed into tobacco, but this did not affect the Si transport, which suggested that a different set of genes might be present in higher plants [91]. The first silicon transporter i.e., Lsi1, was reported in rice root plasma membranes, is a member of the NIP subfamily of plant aquaporin-like proteins [26]. This was followed by the identification of an efflux transporter i.e., low silicon 2 (Lsi2) [28], which revealed that plants use influx (Lsi1; channel-type passive transporters), as well as efflux transporters (Lsi2; active transporters) for the effective transcellular transport of the Si. Detailed studies on homologs of Lsi1 in different lineages of plants have confirmed that the amino acid sequence is well conserved, has six transmembrane domains, two Asn-Pro-Ala (NPA) motifs, and an aromatic/arginine (ar/R) region with the latter two being associated with the substrate selectivity [92]. This unique selectivity filter is a characteristic of the NIP III subgroup (containing all Lsi1 genes from different plants). A site-directed substitution mutation from serine to isoleucine at the H5 position (GSGR to GIGR) resulted in a complete loss of Si transport activity of OsLsi1. However, it does not mean that other transporters such as the boric acid transporter of Arabidopsis (*AtNIP5;1*) can transport Si if their selectivity filter is modified to Lsi1 type [93]. This suggests that some other features of silicon transporters/NIPs might also play a role in Si selectivity. In this regard, Deshmukh, et al. [94] studied the selectivity filters of 25 sequenced plant genomes and discovered that, in addition to the amino acid sequence of the selectivity filters, a precise distance of 108 amino acids between the NPA domains is critical for Si selectivity. While the inter-NPA residue stretch is essential for Si selectivity, the GSGR selectivity filter might also contain other residues. In the horsetail, the selectivity filter contains STAR residues instead of GSGR ones [95]. Contrary to Lsi1, the efflux transporter i.e., Lsi2, which transports Si out of plant cells, has 11 predicted plasma membrane domains and it belongs to an uncharacterized putative anion transporter family. The expression, tissue, and cell specificity of their polar localization varies in plant species and it is important for Si accumulation [12] (Table 1). A total of 62 silicon transporters have been reported in different plants based on a genome-wide exploration. Interestingly, this study concludes that there exists a positive correlation between sequence similarity and the Si accumulation ability of the studied plants, mentioning that the selectivity filter and degree of sequence homology could act as a promising signature to predict Si accumulation in plants with unknown Si accumulation capacity [96]. Apart from these two types of silicon transporters, other Si channels i.e., EaNIP3e (*EaNIP3;1, EaNIP3;3, EaNIP3;4*), have been reported in horsetail. These horsetail Si transporters are permeable to silicic acid, but they harbour a distinct S, Thr (T), Ala (A), and R selectivity filter [95]. Another important study [36] reported that the variation of Si transportability between different cultivars of the same species could arise due to mutations, leading to the mislocalization of influx transporters, rendering reduced Si intake from an external source to roots. The amino acids that were located in the membrane domain (amino acid at position 242) and in loops (amino acid at location 75) of the proteins were responsible for the mislocalization of silicon transporter in the pumpkin to the endoplasmic reticulum. Apart from silicon transporters some studies reported serine-rich proteins that are involved in Si absorption, however the complete characterization of the reported protein is still missing [97]. Proline-rich protein (PRP1) in cucumber has been reported to be involved in preparing the cell walls for enhanced silica deposition at the site of attempted the penetration of fungi into epidermal cells. This PRP1 has C-terminal repetitive sequences having a high amount of lysine and arginine residues [63]. A recent discovery of SIp1 protein in sorghum (*Sb01g025970*) might change the landscape of future research, while the detailed investigations have focused on the discovery and characterization of silicon transporters and silicon transporters-like genes in plants. This protein is a member of the proline-rich protein family and there is only one copy of the SIp1 gene present in sorghum. Only 14% of the protein is alpha helix and it forms a disordered tertiary structure. It is interesting to note that the SIp1 protein has limited sequence homology with other known biomineralization-related proteins. The SIp1 protein has seven repeat units that are rich in lysine, histidine-aspartic acid rich regions, and several proline residues [69]. Four rice transporters (*OsLsi1, OsLsi2, OsLsi3*, and *OsLsi6*), a sorghum *SIp1* gene, and the respective proteins are shown in Figure 2. 

## 4. Shape, Size, and Chemistry of Phytoliths

Aided by water transport and evaporation, the plant systems polymerize/accumulate and they deposit silica in solid form creating intracellular and extracellular phytoliths [101]. The phytoliths are formed in roots, stem, and leaves (and also bulbs, corm, tuber, etc.) of the plants under normal physiological conditions and survive death and decomposition of plants. This biogenic process of phytolith formation creates intriguing irregular (dumbbells, saddles, bowls, boats, bulliform, tracheid, polylobate, etc.) to regular shape (spherical, globular, cylindrical, hexagonal, cubical, and hair-cell, etc.), which may or may not cross different levels of taxonomic boundaries [20]. A single plant species (and sometimes even different plant parts of the same species) can produce many different morphologies of phytoliths [102], challenging phytolith analysts and inviting cross-disciplinary research i.e., involving botany, taxonomy, systematics, plant physiology, chemistry, ecophysiology, ecology, biogeochemistry, geoarchaeology, and palaeontology, establishing it as a research in a super-discipline of earth and life sciences [2]. Such involvement of interdisciplinary researchers may lead towards the development of inconsistencies in the application of different names, synonyms, and homonyms, making the exchange and interpretation of data difficult. Due to this reason, a discussion on phytolith nomenclature aroused during the 3rd international meeting on phytolith research (IMPR) in Bruxelles (August 2000), which later ended up in the development of standardized nomenclature, titled the International Code for Phytolith Nomenclature 1.0 [7]. 

**Figure 3:**
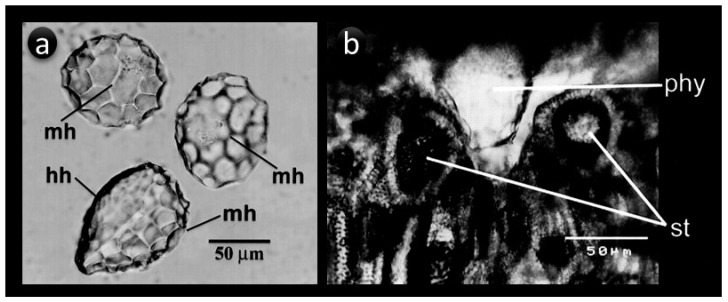
(**a**) Scalloped phytoliths of *Cucurbita moschata* with different rotations showing scalloped impression created by hypodermal (hh) and mesodermal (mh) cells, (**b**) SEM micrograph of *C. moschata* showing interlocked stone cells (st) and phytoliths (phy) (with permissions from [103]).

The particular shape of phytoliths within plants is determined by two factors i.e., the type of cells, where silica is being accumulated, and its precise location within the plant body. However, this case may not be always true, as some of the cell lumens are silicified incompletely conforming the notion that phytolith shape not equivalent to the cell shape [1]. Some phytoliths may develop within the plants with scalloped surfaces, which might be influenced by surrounding cells and have diagnostic features, such as those that are found in rinds of Cucurbita fruits or the bulliform phytoliths in wild and cultivated rice. The bulliform cells in upper epidermis shape the phytoliths while the adjacent colourless cells define the fish-scale diagnostic decorations in rice leaves [103,104]. Articulated dendritic phytoliths formed in inflorescence bracts of many monocots take the shape of the lumen of long epidermal cells, while silica cell, short sell, rondels, and short trapezoid phytoliths in wheat and barley are shaped by the lumen of epidermal short cells. Similarly, prickle, trichomes, long trapezoids, dumbbell, and saddle phytoliths are also shaped by the lumen of respective cells [105,106,107]. A study by Carnelli, Theurillat and Madella [107] surveyed the phytolith types in subalpine-alpine plant species of the European Alps and described in detail the shape of the phytoliths in regard to the cells. The study concluded that monocotyledons mainly yielded phytolith types of epidermal origin, while dicots yielded phytoliths of silicified epidermal jigsaw cells, stomata complexes, and vessels, and the conifers-distinctive conifer cells were mainly silicified. The grass phytoliths that were produced in the epidermis are of two major types i.e., those that formed in long epidermal cells and the others formed in short epidermal cells. Amongst the two types, the latter is much distinctively classified and tend to have subfamily or tribal characteristics [106]. Regarding the control of morphology and size, one might also think of genetic factors behind this, for example, the increased size of papilla phytoliths in genus *Triticum* was thought as a result of ploidy level increase from AA to AABB to AABBDD [108]. In Cucurbita fruits, a genetic locus, hard rind (Hr), has been associated with the spherical scalloped phytolith morphology in the rind. The interlocking of phytoliths and stone cells in the hard rind of Cucurbita fruits restricted the phytolith size and left marks on their surface as scalloped impressions of different sizes (based on the sizes of hypodermal and mesodermal cells surrounding phytoliths) (Figure 3) [103]. The glume architecture locus in teosinte, called teosinte glume architecture1 (*tga1*), was also reported to govern existing distinguishable phytolith assemblages in maize and its wild ancestor teosinte’s fruit bracts [109]. The tissue or compartment where silica is being deposited may also play a role in shaping the phytoliths, for example, silica deposited between the cell wall and cell membrane might also define specific phytolith morphology. The role of cell wall polymers on the morphology of deposited silica is also actively discussed now [110].

**Figure 4:**
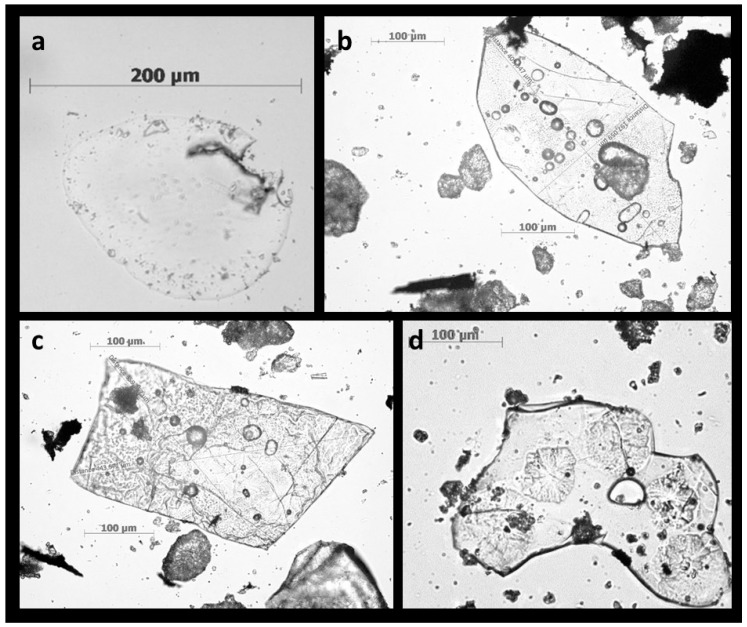
Large sized phytoliths. (**a**) *Cymbopogon schoenanthus* vine, (**b**–**d**) *Melanoleuca grammopodia* (figure source [10]).

Typical phytoliths may vary in dimensions, and their sizes may range from 100 nm to 200 mm [106,111,112,113,114,115]. Some studies show images where the phytoliths are seen, like broken structures, which suggests that the original size of some of these phytoliths may be even larger. These anomalies were observed by our research group, as we found Si structures of up to 500 μm [10] (Figure 4). Interestingly, on the surface of those large structures, imprints of internal plant structures were also recorded, while it remains unknown how these imprints were printed onto the large phytoliths. This might be due to the fact that cellulose microfibrils and other carbohydrates are structures with fixed geometry and order, which may imprint this order on crystal matrix, giving rise to birefringence (Figure 4d) [4,116]. However, the absence of reports on the large-sized phytoliths might be explained by the fact that these large-sized phytoliths can fall out of sight of scientists or by a constant methodological error during phytoliths isolation that originates due to a repeated re-suspension [10]. Recent studies on changes in phytolith size and content have delineated the effect of certain factors, such as growing conditions, age of tissues, and biotic and abiotic stresses. Research on common reed (*Phragmites communis*) has revealed that temperature and precipitation were prominent factors that influence the size of short cell phytoliths, including rondel and saddle morphotypes. The width-to-length ratios of the short cells did not show any variations in response to the differences in temperature and precipitation, and it was suggested that the phytolith size to a larger extent remains under the genetic control [5,117]. In guinea grass (*Leymus chinensis*), the phytoliths originated from long epidermal cells and from intercellular spaces that have been found to be sensitive to CO_2_ concentrations, warming, and nitrogen supplementation [118]. Apart from these factors, another important observation was the differences in the habitat, which influenced the lanceolate phytoliths and the silicified stomata [119]. A recent study by the same authors described that phytoliths are sensitive to warming and the size of saddle decreased, while that of hair cells and silicified stomata increased in response to warming and these responses were variable during different periods of the year in the same plant [120]. Similar results that the phytolith assemblages vary throughout the growing season were observed in leaves of bamboo (*Dendrocalamus ronganesis*) [121]. The diameter of scalloped phytoliths in *Cucurbita pepo* Var. Texana (wild gourd) has also been reported to be affected by pathogen stress (bacterial wild disease), which suggested a significant role of biotic stresses could at least play with the size of phytoliths [122]. Taken together, it could be reiterated that phytolith size is under genetic control and it is variably affected by temperature, heat stress, physiology of the cells, age and maturity of the plant tissues, seasonal climatic changes, and disease incidence.

Recent advances in image analysis and statistical techniques have improved our ability to identify species based on phytoliths that are found in archaeological samples, but it could be sometimes misleading, as phytoliths are formed in different environments in the plant, so they may differ in morphology as well they could be chemically heterogeneous [6]. Phytolith chemical composition coupled with morphology can be used as a proxy to determine the environmental conditions under which the plant was growing [4]. Phytoliths are deposits of amorphous silica and they have the general formula [SiO_n/2_(OH)_4n_]m, where n = 0 to 4 and m is a large number [51]. The chemistry of the phytoliths strongly depends on the chemistry of the environment, such as plant taxa, the composition of the soil, the climatic conditions, pH, the temperature, and the location within the tissue where these are being deposited. Hodson [4] suggested that the two types of phytoliths i.e., those laid out on the carbohydrate matrix and the ones that are deposited within cell lumen, must be different in terms of their composition other than amorphous silica i.e., organic content. The presence of organic matter in each type of phytoliths would define their ability to differently breakdown. Among different fractions of organic matter occluded in the phytoliths, PhytOC is an organic C fraction that is entrapped within phytoliths. This PhytOC fraction is highly stable and resistance against decomposition and remain deposited in the soil for thousands to millions of years, and hence plays a major role in terrestrial biogeochemical carbon sequestration (for the role of PhytOC in C sequestration please see [123,124]). The PhytOC could reflect the environmental conditions and CO_2_ concentrations of the prehistoric time when those phytoliths were formed. However, it is also possible that plants growing in some location might also absorb the organic matter that originated from previous deposits and it could reflect redundancies in describing palaeoenvironment. This has also been investigated in sorghum where plant growth conditions have been associated with the alteration of PhytOC [125]. There has been a number of reports suggesting the presence of elements other than Si and oxygen in phytoliths. For example, the bamboo opal segregations included K, Mg, Na, and Ca, woody species may have Al, and Sc, Ti, V, Cs, Fe, rare earth metals, and depleted major inorganic elements in barley [126,127,128]. X-ray microanalysis among different taxa suggested that woody species produce high proportions of aluminosilicates and this compositional difference from that of herbaceous species’ phytoliths would be an important signature for palaeoenvironmental studies [127]. The elemental composition of barley revealed that the major portion of elements that were present in phytoliths belonged to terrigenous elements i.e., Al, Sc, Ti, V, Cs, and Fe, and most of the contents that were determined from stem represented the endogenous levels of these elements. A variation in the elemental composition could also arise due to contamination present on the surface of the tissue being analyzed, as was observed in the leaves and awns in wheat and barley [128,129,130]. The phytolith composition may also be different in different species growing in the same environment and soil. These observations suggest that geochemical conditions reflect the elemental composition of the phytoliths. Soils in mining areas, polluted soils, and unpolluted soils tend to differ in the elemental composition, and so are the phytoliths deposited in plants growing on these types of soils [131]. 

Post depositional environment is another important factor when considering the chemistry of phytoliths. It is important to note that the phytoliths’ stay in the soil for longer times after plant death and decay may affect dissolution ability (again based on its chemistry), as well as the deposition environment. Different studies have proposed that the presence of impurities such as Al might be responsible for the phytolith stability, however the role of Al in this regard is not yet fully understood. This higher stability might be due to the strong absorption of Al^3+^ in soils and formation of Si-O-Al-O-Si bonds, which reduces Si release [132]. Apart from impurities, the maturity of the phytoliths morphotypes has also been proposed to be related to phytolith preservation. Other factors, such as pH of the sediment, plant taxon, and the conditions of the weathering process have also been affiliated with the stability of the phytoliths [127,133]. Furthermore, under alkaline-pH conditions, they can dissolve and abrasion by mechanical disturbances might also damage the delicate appendages of phytoliths. A differential pattern of stability was observed when modern phytoliths from the wheat inflorescence, stem, and leaves were compared after treating with alkaline pH (pH 10) and burning in an oven [134]. Liming enhances the dissolution of phytoliths through increasing the pH [135]. Phytoliths may partially dissolve under variable depositional environments, depending on their surface area to bulk ratio. Cabanes and Shahack-Gross [133] suggested that “the lower the solubility of a given phytolith assemblage, the more it is composed of resistant phytoliths”. The authors classified the assemblages based on Si solubility values i.e., values below 2 mM are highly distorted morphotype compositions and poorly preserved. Whereas, values between 2–3 mM show good preservation state, while values above 3 mM are excellently preserved with slight or no distortion. Studies on pretreatment and solution chemistry on the dissolution of phytoliths have demonstrated that the presence of different cations and anions and their concentrations in the soil coupled with pH affects the phytolith dissolution. Burning of rice straws at 400 ℃ also yielded phytoliths and biochar in the ashes. These findings suggested that monovalent cations (possibly due to high ionic strength) highly suppressed Si release when compared to bivalent cations. These findings are very important for Si availability for the next growing season after burning the rice straw [132]. The isolation method may also sometimes lead to confusion in understanding the chemistry of phytoliths, as unknown contaminants were observed due to inefficient acid digestion of organic material in barley [128]. The plant tissue to which silica is being deposited may occasionally lead to the presence of contamination. It could be seen in phytoliths growing on the cell wall that may have carbohydrate and protein impurities, while those deposited in the lumen may contain breakdown products from the protoplast. Large amounts of sugars and/or proteins in water-soluble form and lipids in an insoluble form, as well as degraded glycol-conjugated proteins have been observed in modern wheat phytoliths [136]. The authors also hypothesized the presence of ancient DNA that was trapped within phytoliths and employed a hypersensitive essay to determine the naked DNA. However, there was no indication of DNA found within the phytoliths, leading towards the understanding that either the DNA was absent or was not recoverable. 

## 5. Evolution of Biosilicification in Plants

Biosilicification is a process that crosses kingdoms of living organisms and it is not strictly restricted to plants. Silicification is present in different lineages i.e., stramenopiles, rhizarians, opisthokonts, testate amoeba, and plants (particularly the land plants). The involvement of Si in calcification and bone formation extends the scope of biosilicification research and the search for silicon transporters has led to the identification of genes in eukaryotes and prokaryotes [137]. Marron, et al. [138] suggested that silicon transporters evolved independently or via horizontal gene transfer that was based on the fact that they are present in taxonomically isolated lineages. Later, it was discussed that eukaryotic last common ancestor may have possessed silicon transporters pointing towards an ancient deep origin in eukaryotic phylogeny [109]. Among plant lineages, grasses have a special position in the global biological silica cycle, due to their ability to accumulate higher amounts of silica. However, grasses are not the only high Si accumulators but many members of early plant lineages have to accumulate relatively higher Si amounts as compared to grasses e.g., *Sellaginellacae*, *Equisetacea*, *Marattiacaeae*, and *Osmundaceae* [139]. Horsetail is quite a familiar example of substantial Si accumulator; an early diverging fern. Katz [140] suggested that grasses, commelinids, and aquatic macrophytes (Si accumulators) have evolved Si accumulation as a strategy to defend themselves against herbivory and abiotic stress, but still they do not represent a complete picture of angiosperm silicification diversity. The silicon accumulation ability has also been reported to differ based on intra-species differences [94]. Evolutionary adaptation of silica accumulation in plants in the form of phytoliths is still an active topic, and the origin of this trait is unknown, even with the availability of large scale whole genome sequences of various plant lineages and abundant data of silica accumulating potential of modern and early plants (preserved as fossils). The fact that bacterial genome contains NIP-like proteins suggests an evolutionary consequence that this ability to transport Si may have been transferred to early terrestrial plants via horizontal gene transfer from bacteria e.g., the presence of FAAR NIPs in mosses [141,142]. Once transferred into plants, these ancestral aquaporins subsequently evolved (neofunctionalization) into other NIP clads through evolutionary forces i.e., duplications and mutations. Marron, Ratcliffe, Wheeler, Goldstein, King, Not, de Vargas and Richter [100] proposed that silicon transporters evolved in earlier plant lineages to cope with Si toxicity in high Precambrian oceans, and they were lost (silicon transporters and silicon transporters-like genes) in Phanerozoic seas due to biologically induced reductions in Si concentrations. However, rare observation of NIPIIs in gymnosperms; despite a large number of fully sequenced plant genomes, the loss of silification ability in the last common ancestor of seed plants and the presence of NIPIIIs in angiosperms may be due to a secondary gain of function [139,143]. Mapping analysis by Strömberg, et al. [144] also suggested silica accumulation in plant lineages multiple times. Furthermore, Strömberg, Di Stilio and Song [144] considered multiple evolutionary questions to consider whether or not the phytolith emerged on the basis of a solution to a problem i.e., for various structural and resistance mechanisms that were related to plant ability to withstand various biotic and abiotic stresses [145]. However, the patterns that were observed in their study suggested that this adaptation/acquirement might not be evolved in basal angiosperms, but as independent acquirement in various sub-clads; per se need based acquirement [146].

## 6. Functions of Phytoliths

### 6.1. Biotic and Abiotic Stress Tolerance

Silicon is not an essential nutrient in plant growth and development, but it has beneficial effects. The role of accumulated Si in the form of phytoliths as well as in other forms influence many aspects of biology by improving plant fitness in nature and increases agricultural productivity. The difference in the amount of Si that was deposited in different plants gives them different abilities under different biotic and abiotic stress [147]. Mainly, the role of Si accumulated in different cells and organelles i.e., cell wall, exodermis, and endodermis as phytoliths is to act as a barrier, rather than playing bioactive roles. Silicon can act as an activator of plant disease resistance by playing an active role in inducing disease resistance i.e., system acquired resistance (SAR). This SAR must be dependent on an inducing compound i.e., salicylic acid, which is locally accumulated at the site of infection and systematically to be activated. However, Si as an activator of SAR losses rapidly its ability for Si mediated resistance when it is exhausted from the nutrition solution [63]. This is due to the fact that, Si, when accumulated/polymerized, loses inactivation as an inducer of resistance [148]. However, the role of Si on the plant is far more pronounced and complex, as it transcends its barriers from cellular level to plant metabolism [149]. Hence, our objective in this section is to present a glimpse of the roles of Si in plants and discuss how the presence of phytoliths helps plants against various biotic and abiotic stresses. 

### 6.2. Water Stress Tolerance

The mechanism by which the Si affects plants under water stress conditions is not explored in many studies and, until recently, it is a topic of great interest. The change in hydraulic conductivity and osmotic adjustment could be one reason and this might be due to the regulation of different genes e.g., under osmotic stress the expression of HvLsi1 and HvLsi2 in barley [150]. In a simulated water stress study on wheat, the Si deposited as a thin layer in the epidermal cells and embedded with trichomes, giving more rigidity to the cells. Furthermore, comparable amount of phytoliths was produced by the plants that were supplied with Si treatment. The silicification was only restricted to silica cells on the veins (Polyethylene glycol treatments) as an indicator of water stress [13]. This change in behaviour might signal that the wheat plants protect veins (i.e., vessels) to keep the water supply running in limited water conditions. The authors suggested that this adaptation might give additional support to plant leaves and better light interception leading towards better photosynthesis. The development of silicified trichomes under stress conditions is controversial, as the silicification was restricted to trichomes in the case of soybean and cucumber, but in the case of wheat the silicified trichomes did not appear, as discussed above [151,152]. Furthermore, this could be due to decreased electrolyte leakage against increasing Si accumulation in leaves [153]. This might be due to the difference in silicification process in dicots and grasses and need further confirmation. With the onset of drought, the plants with higher Si deposited beneath the leaf cuticle adapt to reduce transpiration rate due to the Si-cuticle double layer in rice [23,154]. A study regarding the role of Si in water use efficiency reported that the change in the transpiration rate in response to Si treatment is due to its impact of the stomata movement in maize. Furthermore, it was also suggested that silica deposits also increased water affinity in xylem [155,156]. The higher rate of cell wall silicification in leaves may also be involved in water relations of the cells by improving the mechanical properties and water permeability. 

### 6.3. Salt and Metal Stress Tolerance

With the onset of salt stress, plants undergo ‘physiological stress’, coupled with drought stress. Plants respond to these stress conditions by two different reactive oxygen species scavenging mechanisms i.e., enzymatic and non-enzymatic. The presence of higher amounts of Si in the cellular environment and vicinity help the plant to withstand and resist various stresses that are associated with salt stress [157]. For example, in the change in osmotica contents in an attempt to maintain tissue hydration, plants undergo osmotic stress. Si accumulation amplifies defensive responses, as explained in the above section. The accumulation of proline and free amino acids was increased in response to increased Si treatment, which suggested the role of Si in osmotic adjustments by inhibiting lipid peroxidation [158]. Both rice Si transporter genes showed higher expression under salt stress in addition to Si supplementation. Many studies on different plants have suggested that Si prevents the adverse effects on salinity on the plant by avoiding Na^+^ uptake in the plant roots and its transport to shoots [159,160]. This mechanism behind the control of the levels of Na^+^, as well as K^+^ under salt stress conditions by Si, might be due to its stimulating effect on H^+^-ATPase and H^+^-PPase. However, this hypothesis needs further exploration [161]. The deposited Si on root exo- and endodermis hinders apoplastic transport i.e., reduction in tranpirational bypass flow and therefore Na^+^ transport in rice, but this mechanism is not universal to all plants and it may differ. Silicon has also been involved in improving water storage in plants and thus making the salts diluted in the cells [162]. Higher concentrations of essential nutrients in tissues of salt-stressed plants suggested that Si has a positive effect on mineral nutrient balance [163,164]. Silicon has been found associated with amelioration of heavy metal stresses. Several mechanisms coordinate to help a plant to withstand heavy metal stress, such as the reduction of heavy metal uptake, changing the pH of soil, formation of compounds by combining with the heavy metals (e.g., aluminium silicate), and the stimulation of enzyme activities (e.g., regulation of P-type heavy metal ATPases) [165,166,167]. Considering the phytoliths as the focus of this review it is important to note that lignin-bound silica in cell-walls improves metal binding and reduces the transfer of metal ions. This happens when silica within cell-walls creates complexes with metal ions and it may also precipitate metal ions as co-factors [168]. Ma, et al. [169] reported that hemicellulose-bound silica with a net negative charge inhibits cadmium uptake in rice by Cd complexation and subsequent co-deposition. The mentioned mechanisms are coupled with the modification in expression levels of silicon transporters most of the time. For example, OsLsi1 and OsLsi2 were up-regulated when the effect of increased Si was investigated in rice cell suspension against cadmium and Al stress [168,169]. Similarly, the expression of both rice Lsi genes was significantly suppressed in response to the application of glycine to reduce arsenic accumulation in rice seedlings [170]. Furthermore, the mineral nutrient uptake and biochemical response of seed priming with Ar-Si is also modulated by changes in the Lsi gene expressions in rice [171]. Apart from silicon transporters, it has been established that Si application also effects on the expression of genes that are involved in the transport and allocation of nutrients, as well as heavy metals [172]. High Si concentration down-regulates the phosphorous-transporter genes [173], genes related to Fe acquisition mechanisms (*MsIRT1* (Fe transporter), MsNrampl (metal transporter), and *OsFRO1* (ferric chelate reductase)), whereas the expression of Alfalfa metalcheloators (*MsPCS1* (phytochelatin synthase), *MsMT2* (metallothionein) [174]. It also induces antioxidative responses and the expression of boron transporter (BOR2) and members of aquaporins (PIP1 and PIP2) [175]. These findings stress that cross-talk between expression (upregulation and downregulation) of silicon transporters and different metal transporters in response to Si application and heavy metal stress should be further explored. 

### 6.4. Tolerance to Pathogens

The functional role of phytoliths deposited in the cell-wall matrix as a barrier against pathogen entry in the plant cell has been well studied [145]. The main concept behind this protection is that the cell walls in rice and other plants are reinforced with Si and they enhance pathogen resistance. This hypothesis is further supported by the fact that Si has been found to be accumulated on the infection sites of various pathogens, as observed in Arabidopsis and rice, respectively [176,177], but it is thought that this Si accumulation at the infection sites is actually attributed to higher transpiration; at damaged cuticles, rather than a defensive mechanism [178]. This tolerance should continue even if the Si supply is stopped because the plants must have deposited silica/opal in their cell wall matrices and should continuously act as a barrier but in an experiment by Samuels, et al. [179], the opposite was observed. This suggested that the accumulation of Si in different locations as a barrier should not alone be responsible for pathogen resistance. Instead, there exists a role of Si in activating the defense mechanism again pathogens. Enhanced activity of many enzymes i.e., chitinases, poyphenoloxydases, and peroxidases, coupled with higher flavonoid phytoalexin production, has been reported in (Pythium and powdery mildews) infected dicot plants [180,181]. However latter studies confirmed the similar mechanisms of an active role of Si in plants against pathogen attack. To induce resistance, silicon interacts with several key components. For example it can act as a secondary messenger in induced systemic resistance by binding to hydroxyl groups of different proteins involved in signal transduction. It is also known to interact with cationic co-factors of enzymes that are involved in pathogenesis-related events [178,182,183]. 

### 6.5. Role of Phytoliths against Herbivory

Phytoliths in plants serve a variety of purposes. Dental microwear of mammalian grazers due to phytolith presence in plants has been considerably discussed in the literature, mainly due to the concept that phytoliths are harder than mammalian tooth. Most of the mammals, particularly grazers, eat grasses that are known to be Si accumulators as well as are characterized a high density of parallel linear striations [184]. Since this publication, there has been intense debate on the tooth wear, as it is directly related to mammalian life expectancy. The aberration that was caused by hard silica phytoliths on the tooth is mainly referred to the study by Baker, et al. [185], who compared enamel from sheep with silica phytoliths from oats. However, a recent study showed that silica phytoliths from four grass species are softer than sheep dental enamel (51–211 Vickers Hardness (VH) versus 293 and 375 VH, respectively), implying that phytoliths are not a predominant cause of tooth wear in sheep and mammals [186]. Accordingly, toothwear in grazers is not limited to phytoliths and it could also be due to abrasive covering ingesta and tooth tissue chips. Lucas, et al. [187] compared the properties of phytoliths, grit, and dust, and suggested that it is very unlikely that phytoliths may wear enamel, but somehow they (phytoliths) resemble natural agents of wear and tend to fracture between teeth under natural conditions. Some studies indicated higher Si accumulation in plant leaves in response to grazing [188,189,190] and suggest this a strong mechanical defense mechanism against herbivory. Dental microwear texture analysis on lab voles fed with pelleted diet with differing amount of Si resulted in similar dental textures. Contrastingly, dental microwear in wild voles was proposed to be the result of grazing-induced phytolith concentrations, however further confirmations are still needed [191]. Another study showed that the evolution of dentition in horse is correlated with variation in phytolith content of grasses and preference of grazing C3 grasses over C4 grasses [192]. This hints towards two different mechanisms, where the first is the toothwear in herbivores and the second is the taste of the grasses. Higher accumulation of the phytoliths in plants makes them distasteful, as well as gives them a prickly texture, which grazers usually avoid [20]. This could also be the reason for older seedlings to be better defended, owing to the higher accumulation of phytoliths [193]. The higher amount of deposited Si in plants hinders the absorption of nitrogen from digested plant material in the mammalian body. This reduced nitrogen absorption has been linked to a reduced growth rate of voles; this could suggest that Si in plants can contribute towards the cycle dynamics of vole populations [194]. It is worth noting that Si content and accumulation within a plant cannot be a direct measure of mammalian avoidance of grazing, but many other factors, such as specie of mammal and plant, environment, forage quality, and types of soils, may also contribute in this regard. Another important, as well as an interesting, factor is the presence of endophytic fungi living on the grass species. These fungi have different abilities to produce mycotoxins and they can reduce the palatability of grasses [195]. Taken together, plants synergistically employ three potential defensive mechanisms i.e., Si induction, endophytes, and secondary metabolites [196]. 

### 6.6. Role of Phytoliths as Mechanical Barriers

The beneficial effects of Si in plants have been mainly discussed for its role in defense mechanisms against biotic and abiotic stresses as a non-essential element [23]. However, in this section, we will discuss the role of phytoliths as mechanical barriers to various types of stresses. In above sections, we have explained how silica is deposited in plants in spines, trichomes, and beneath the cuticle in leaf blades and in cell walls. The presence of Si in the cell wall ultimately leads to rigid stems and leaf blades giving plants mechanical support against lodging. The chemical composition of cell wall i.e., cellulose, lignin, deposited silica, and the structural carbohydrates in the basal node determine the stem rigidity and breaking resistance in cereals [197]. Higher rigidity by employing more silica deposition is 10–20 times more cost-efficient for plants when compared to carbon-based alternative, and hence Si is given competitive advantage to plants and increases their primary productivity [198] The silica phytoliths and cell wall polymers, together protect cell membranes from structural and functional deterioration induced during abiotic stresses [199]. At higher temperatures, the thermal stability of membrane lipids is protected by phytoliths; the electrolyte leakage is reduced [153]. The cell injury caused by low water during the onset of osmotic stress may shrink the cells and damage the cellular machinery; however, deposited Si along with cuticle prevents the excessive water loss for the plant [23,154]. Structural stabilization of cell wall with Si is high, and it can delay the senescence of the potato tuber skin by increasing suberization [200]. The deposition of Si is related to cell wall polymers, but, during Si deprivation, the cell-walls experience a lack of SiO_2_ and signals cell-wall thickening, leading to decreased mechanical strength [201]. This reduced mechanical strength allows for pathogens to attack and penetrate plants easily [110]. The accumulation of silica in leaves that live longer was previously considered detrimental for photosynthesis however, this was nullified in small bamboo [202]. The accumulation of Si in plant tissues increases the rigidity and stiffness and silicified cells, in turn, provides resistance against culm breaking i.e., lodging resistance. This resistance is an overall ability of Si to improve lignin content, higher silica accumulation in cell walls, and the formation of silicified microstructures [203]. This mechanical strength has also been shown to be affected by the interaction of phosphatidic acid with Lsi6 in nodes [204]. The rate of photosynthesis has been positively correlated with higher Si accumulation as observed in cultivated strawberry (*Fragaria×ananassa* ‘Camarosa’) [205]. The increased photosynthetic ability of plants is mainly two reasons i.e., first because the phytoliths increase leaf erectness and hence providing large surface area to absorb more sunlight [13] and the added silica increases sunlight interception. Apart from sunlight, the protective effect of leaf epidermis deposited silica is also important in UV radiation absorbance; the increased Si supplementation in rice resulted in absorbance of less UV [206]. Higher Si accumulation in leaf has also been shown to alleviate the adverse effects of UV-B. Increased total biomass, chlorophyll content, soluble sugars, and phenolic flavonoid compounds is also a positive effect on silica deposited in leaf [206,207]. The excessive transpiration from silicified trichomes may contribute to cooling the plants, but it has not been extensively studied [208]. 

Apart from functions of Si that are discussed above, many recent studies have demonstrated that (1) it stimulated high nutrient uptake such as inorganic phosphorous (Pi) uptake increased by many fold upregulation of Pi transporters in wheat i.e., *TaPHT1.1* and *TaPHT1.2,* as well as by increasing malate and citrate exudation rates [209]. (2) It also promotes plant growth by improving plant biomass, increases nitrogen uptake, upregulates the nitrate transporters, increases photosynthetic activity, decreases oxidative stress, and delays senescence [210]. (3) Under combined deficiency and osmotic stress, Si nutrition transcriptionally regulates the sulfur and abscisic acid metabolism, promotes cytokinin biosynthesis, and delays leaf senescence [211,212]. (4) Silicon enhances the expression level of the Strategy II Fe uptake related genes in roots and upregulates the gene expression in leaves of Fe deficient barley [213]. (5) Higher Si concentrations in leaves are linked with short-lived leaves, which suggests that leaves consider Si as a cheaper alternative to carbon and allows for more favorable leaf carbon balance over short periods [214]. 

### 6.7. Phytoliths as Taxonomic Tools

Phytoliths persist thousands to millions of years in a variety of soils, owing to their hard and resistant morphological features, shapes, and chemistry. Phytoliths are preserved with and without carbonization, and hence help scientists to explore complementary data about flora that might have been affected by decompositional processes before and after deposition on the site. These different and unique phytoliths produced by different species can provide clues for taxa identification in the taxonomic hierarchy. For the past few decades, exponential growth in phytolith analysis in archaeological sites and modern flora has been proven useful in identifying plant dispersal, domestication, agricultural practices, and human and animal feed in past civilizations [144,215]. Here, we summarize the role of phytoliths as taxonomic tools and reconstruction of information on ancient flora, environment, and agricultural practices. We aim to present as many functions of phytoliths rather than piling up examples of similar nature, owing to a huge amount of studies. 

#### 6.7.1. Identification of Genus and Species

An abundance of phytoliths in vascular plants has proven to be diagnostic feature for the differentiation of genus and species. Distinct phytolith shapes and sizes have emerged as descriptive tools and proxies if these are exclusively found in the specimen under investigation [7]. Among many comprehensive reports on phytoliths, the report by [216] on the new world plant species, including wild and cultivated species of non-graminaceious plants and by Kealhofer and Piperno [217] on the distribution and morphology of phytoliths in 77 families and 377 plant species were the very first reports. The distinction between some species sometimes makes it challenging due to the occurrence of similar morphotypes in different plant species. Morphometric analysis of phytoliths assemblages of closely related taxa has been proven useful in the inflorescence bracts of *Triticeae* [218]. Some studies prefer finer shape and ornamentation is more reliable descriptors than the size of phytoliths [199]. Morcote-Ríos, et al. [219] surveyed contemporary phytoliths in 92 palm species that represented four subfamilies and 29 genera from Amazon and reported eight distinct phytolith types with a higher degree of taxonomic resolution. The use of fine details as overall geometry, detailed surface ornamentation, including the density and morphology of the dents and peaks on the phytolith surface, has proven useful in the identification of six palm species from sediment samples of pan-Pacific distribution [218]. The application of multivariate analysis on phytolith diameter in identical taxonomic samples of main palm species from subtropical regions of South America proved useful for taxonomic distinction [220]. A study from the Indian subcontinent on *Chloridoideae* grasses revealed that saddle shaped phytoliths are characteristic of this subfamily [221]. Phytoliths produced in inflorescence bracts of the barley (*H. vulgare* and *H. spontaneum*) and durum wheat (*T. monococcum, T. dicoccon, T. dicoccoides*, and *T. aestivum*) proved to be reliable tools for the taxonomic ranking of species [222]. Similarly, foxtail millet (*Setaria italic*) and common millet (*Panicum miliaceum*) identification in archaeobotanical remains was resolved by using key diagnostic phytolith morphological features. This study combined the use cross shaped types, regular arrangement of papillae, Ω-undulated type, ending structures of epidermal long cells, and surface ridgy line sculpture in the species [223]. Another study used a similar methodology to distinguish the foxtail millet from green foxtail (*S. viridis*) [224]. Multiple discriminant analysis of rondel phytoliths have been reported to distinguish between species and sub-species of teosinte and it can be used to enhance our understanding of its use in human and animal diet and its domestication [225]. 

#### 6.7.2. Differentiation between Wild and Cultivated Species

Agriculture was a prerequisite for human populations and the rise of civilizations. Inferring history of the domestication of currently cultivated crops reflects genetic changes, the genius of early farmers, and evolutionary history [226]. It is important to distinguish wild and cultivated plant species in order to understand the domestication history and study the ancient agricultural systems. Phytoliths, along with macro-remains, pollens, and carbon dating, have established themselves as a useful strategy to identify remains of crop plants and their wild progenitors in paleoethnobotany during the last few decades [227]. The history of human societies and the history of crop plants are inseparable. Phytolith can answer these questions by distinguishing wild and cultivated plant species. Many modern methods using modern agricultural systems have been proven to be useful in identifying formations of prehistoric agricultural systems. Analyzing phytolith assemblages from surface soils from wild plant fields, cultivated fields, and non-cultivated fields can be a discriminatory procedure in this regard, as observed in paddy fields in China [228]. Huan, et al. [229] successfully distinguished between wild and cultivated rice. In another study, wild (17.46% ± 8.29%) and cultivated rice (63.70% ± 9.22%) were distinguished based on the proportion of bulliform phytoliths with ≥9 fish-scale decorations [104]. The differentiation of maize (*Z. mays* L) from its wild relative teosinte and other related wild grasses was made possible because of typical cross-shape phytoliths. Wild squash has small scalloped phytoliths, whereas in domesticated species *C. moschalta* the size is relatively larger [1]. Heavily silicified elongate dendritic cell forms are distinctive between cultivated and wild sorghum, as these cell forms appear in some quantity in domesticated sorghum, but they are rare in the wild [230]. A good work on understanding agricultural origins of crops and dispersals round the world by employing phytoliths is available elsewhere [227]. Ball, Gardner and Anderson [222] used phytoliths as reliable tools for distinguishing between different wild and cultivated wheat and barley species. 

#### 6.7.3. Historic Uses of Plants by Humans and Investigating Cereal Economies

Phytoliths are a tool for investigating a cereal economy by identifying grass families at archaeological sites, as observed in Po plain in northern Italy (The Terramare civilization ca. 1650–1150 cal BCE). The archaeological context outside the domestic structures is an important application of plant silica casts. Phytoliths from settlement waste of the Bronze Age site in Italy gave many clues towards the use of chopped fodder and processing of cereals, such as small hulled millets [231]. Another study by Dal Corso, et al. highlighted the application of phytolith analysis to the site Maidanetske (ca. 3900–3650 BCE) in central Ukraine and laid out an interesting picture of site subsistence economy [232]. The inseparable information regarding the history of human societies and crop production systems has been resolved by phytolith evidence, as in case of domestication event of rice 9400 cal yr B.P. in the lower Yangtze Basin during the beginning of Holocene [233]. Phytoliths can reveal integrative information regarding cereal and grass usage for food and non-food purposes, such as thatching, making household items for food storage (basketry), temper the small earthen construction, roofing, and matting the indoor house floors [234,235]. Another application is to understand the production systems of a proto-historic agrarian society. As most of the phytolith investigations include *Poaceae* phytoliths, it is understandable that many studies only focus on grasses. Furthermore, as the main plant diets of humans have been members of *Poaceae* (wheat, rice, barley, millets, etc.), so culms, leaves, and inflorescences which biogenerate distinct silica casts, open up a wealth of novel applications to understand human activities in agricultural systems and agro economies [215]. Another useful dynamic of phytoliths is the identification of ancient water availability. This is, in turn, linked with the amount of transpiration in arid and semi-arid regions; vegetation in these regions have a high transpiration rate and thus higher Si uptake and deposition [236]. Many studies used the ratio of fixed to sensitive phytolith forms as a proxy to water availability. In turn, such ratios can give a hint of the irrigation conditions that were prevalent in the pre-historic agricultural systems [237]. One such example is the study that was conducted by [228], where they predicted the growing conditions of rice during the Neolithic in the Lower Yangtze valley in China. In a similar follow up three-year study on durum wheat and barley confirmed the effectiveness of using phytoliths as proxy to estimate ancient water management [238]. The domestication and crop diffusion of bananas in archaeology have relevance with the phytoliths that were produced by the true bananas belonging to genus Musa and help researchers to predict how people managed plant resources in tropical forests and whether these plants were cultivated outside Asia or not [239].

#### 6.7.4. Reconstruction of Ancient Floras, Landscapes, and Palaeoenvironments 

The role of phytoliths in understanding ancient floras and environments recently gained attention, as this earth-life science discipline involved more and more scientists from different fields [2]. The unrecognized history of vegetation in the tropical savannah region of north Western Australia helped to reconstruct the vegetation pattern and climatic conditions by analysing phytolith assemblage from the archaeological site of Carpenter’s Gap 1 [240]. The use of reedgrass (*Phragmites australis*) by Philistines and their daily life during the Iron Age was investigated by phytoliths that were recovered from Tel Miqne [241]. Fossil phytoliths from Pinnacle Pint caves 13B and 5/6 were investigated to reconstruct the environmental conditions and changes in vegetation. During the Middle Stone Age, the C3 grasses dominated the area with the occupation of C4 grasses during specific occupation periods [242]. The reconstruction of palaeoenvironment and vegetation landscape in the Middle Stone Age at southern Rift Valley of Africa was made possible with the phytolith analysis in association with other data obtained from sedimentary, micromorphological, artefactual, and optically stimulated luminescence [243]. Nature of human occupation, reconstruction of palaeoenvironment, and understanding of the climate and vegetation surrounding the cave during Middle Palaeolithic to Neolithic was achieved by using phytoliths from Palaeolithic cave of Theopetra, central Greece and pointed out the intensive occupation of the cave when penultimate glacial was transiting to interglacial period. Every day anthropogenic activities could be understood by identification of specific morphotypes of phytoliths of different plant origins; different plants were used for food, feed, fuel, and other activities during the last 140,000 years at the site [244]. To achieve more detail on the sub-environments, phytoliths of grass and non-grass vegetation have already been used to discriminating coastal and deltaic sub-environments [106,245,246].

Dominant flora during ancient landscape also provides details regarding the climatic conditions, human activity, and major environmental events. Many studies used phytoliths as proxies to reconstruct the landscape of flora [247,248]. Modern soils and prevailing plants can help in such landscape reconstruction. Modern soils from Serengeti National Park, Lake Eyasi, and Manyara and the palms still present there were studies through phytolith analysis and found that sometimes the freshwater courses may influence the number of phytoliths that were preserved in the soils and imply that post-preservation scenario should also be accounted for when reconstructing palaeoenvironments and landscapes [249]. In absence of modern analogues, a phytolith study can be a more reliable and general approach for example, the in early Miocene the modern groups of open-habitat grasses were important vegetation and the north-western Nebraska ecosystems were colonized by a mix of various plant genera, including grasses and dicots, and together made a woodland habitat [250]. Members of *Poaceae* have been prehistorically used by humans for food and feed, as well as are now known as Si accumulators, it is therefore not surprising that most of work on using phytoliths in delineating ancient landscapes has been attributed to grasses [215]. Phytoliths investigations sometimes challenge the previous paleobotanical studies, such as in FLK North and surrounding localities during upper Bed 1 time, has been thought to have mixed opinion about groundwater (either it is fresh or saline) and predominant vegetation (grasslands or mixed C3-C4 vegetation or open to close vegetation). The phytoliths (the site in Olduvai Tanzania) based data suggested that groundwater was not saline and that the region was dominated by palm forest/woodland or bushland in early Pleistocene [251]. 

#### 6.7.5. Discrimination of Forests and Grass Covers and C4/C3 Grasslands

The dominance of forests or grasses is favoured by the atmospheric partial pressure of CO_2_, atmospheric temperature, latitude, light availability, availability of fresh water, frequency of grazers, and environmental disturbances [252]. The reliability of global vegetation models to predict the distribution of grasses is usually less when using ancient to modern day environmental conditions. In this case, phytoliths are suitable proxies to infer past changes in types of grasses or forests/woodlands. So far, many studies have used phytoliths for reconstructing C3 versus C4 grass dominance in many areas, such as North American Great Plains, central Great Plains of Nebraska during Holocene, Columbia Basin, Southern Africa, North America in late Tertiary, and tropical Africa (340,000 years) [253,254,255,256,257,258]. Modern-day vegetation and tree cover density can also be reconstructed and discriminated by using phytoliths with the inclusion of data, such as temperature gradient and elevation [251]. Alexandré, et al. [259] reported that phytolith assemblages from tropical savannas of African lakes could be distinguished as cooler, tall grasses versus warmer, short grasses. Furthermore, the phytoliths also revealed the relative density of trees and shrubs in comparison with cover grasses. Phytoliths from various lake records have been able to discriminate between lowland evergreen versus semi-evergreen forests and C3 versus C4 grasses [217,260]. Piperno [1] reported that C3 dominant grass cover can be differentiated from C4 dominated grass cover, and even within a C4 grass cover the family distinguishing is possible. This concept has been applied to understand the Neogene transition from C3 to C4 grasslands in North America [261,262]. 

#### 6.7.6. Limitations Associated with the Use of Phytoliths as Taxonomic Tools

Phytolith characteristics are as equally important as palynomorphs as well as plant and animal remains. The phytolith production pattern among and between plants, the preservation conditions, and the post-preservation changes should, therefore, be critically considered for the reconstruction of past information on environment, fauna, flora, etc. [6]. Although, the knowledge on phytolith morphology and chemistry has improved our understanding to consider them as reliable taxonomic proxies during the last few decades, still there exist several contradictions and taphonomical variations that invite future research. (1) Phytolith shape overlap between unrelated taxa, which is mainly due to the comparable cell morphology in different tissues. This redundancy could be overcome by improved taxonomic resolution using three-dimensional (3-D) morphology with relatively better resolution. (2) Overrepresentation of phytolith assemblages of grasses and the underrepresentation of low silica accumulator assemblages can sometimes lead us to a much-generalized conclusion of considering the flora just a woody species or grasses [263]. (3) Phytoliths that were deposited in soils/sediments are sometimes transported away from their site of original deposition by bioturbation and illuviation), leading to bias in sampling. This transport combined with different factors i.e., rate of sediment accumulation, initial amount/concentration of phytoliths in deposition sediment, the effect of plant growth (also the plant species), and the effect of root activity on phytolith production and dissolution, is responsible for different preservation states [264]. Radiocarbon dating of stratified soil profiles would reveal more promising and true sample statistics in this regard. It is important to note here that in case of lack of clear sediment-based stratigraphy and less availability of bioturbations may show horizontal and vertical mixtures of phytolith assemblages, and may be erroneous e.g., phytolith assemblages in the core of Izbet Sartah, Israel [265]. (4) Phytolith dissolution, as discussed earlier (see Section 4), is affected by the shape, size, degree of silicification, chemistry, and occluded organic material differs in grass and non-grass species, and creates bias for the reconstruction of ancient non-grass floras e.g., tropical floras [132,133,135]. (5) PhytOC has been assumed to be from atmospheric CO_2_ and lead toward explaining ancient photosynthetic pathways, the age of the mineralized plants, and the terrestrial sink of C in the global C cycle, derivation of paleo-atmospheric O_2_ record through isotopic analysis. However, many studies have demonstrated the differences between δ13C_tissue_ and δ13C_phytOC_ values of different plants, rendering the mentioned derivations erroneous [1,262,266]. These variations could arise due to an older organic matter component being taken up by the plant roots, nitrogen assimilation, or in dissolved form may end up occluded in phytoliths, and this may lead to bias. Hence phytolith purity should be considered for such analysis [267]. (6) Diagenesis and (7) pedogenic silica grains (these silica grains can be superficially similar to some phytolith morphotypes) may lead toward a bias in the interpretation of fossil assemblages [6]. Particularly, phytoliths in shallow sites are relatively more prone to diagenesis with a relatively active soil silicon cycle. (8) Partial phytolith dissolution affect the oxygen and Si isotopic composition of phytoliths (opal-A), as well as (9) the precipitation of new silica can occur.

Based on these limitations, care should be taken during sampling and absolute phytolith concentrations should be determined in the sediments. Sites that are not well preserved and/or that are considered open-air deposits should be dealt with extra care and only phytolith with concentrations of several orders of magnitude should be considered. Phytolith preservation should be evaluated before using them as proxies in paleobiologic reconstructions. The interplay of different factors that are involved in the preservation and post-preservation anthropic signals should be considered. A unique and appropriate count size could be complemented with Phytolith Variation Index to assess the whole viability of the spectra. High-resolution 3-D mapping should be implemented. Samples should be prepared in a way that they should be free from organic material. Phytolith processing protocols and analyses within the laboratory should be improved in order to minimize the potential effects of cross-contamination of samples. 

## Figures and Tables

**Table 1:** A summary of silicon transport related genes characterized in different plants.

Species	Silicon Transporter (Acc. No./Gene ID)	Tissue Localization	Sub-Cellular Localization	Expression Pattern (Si treatment effect)	Spatial Expression	Reference(s)
Channel Type Si-Transporters						
*Orzya sativa*	*OsLsi1*^NSCR^ (Os02g0745100)	Root (Exodermis and endodermis)	Plasma membrane	Downregulated	Mature roots	[26]
*Hordium vulgare*	*HvLsi1* (100301576)	Root (Epidermal, hypodermal and cortex cells)	Plasma membrane	Unaffected	Mature roots	[30]
*Zea mays L.*	*ZmLsi1* (542643)	Roots (Epidermal, hypodermal and cortex cells) Polar Localization at distal size Kernel (pericarp, embryo, endosperm)	Plasma membrane	Unaffected	Immature and Mature roots Immature and Mature Kernels	[14,46]
*Triticum aestivum*	*TaLsi1* (ADM47602)	All tissues of roots and shoots	Plasma membrane	Unaffected	Central Cylinder within the root differentiation zone	[45]
*Cucumus sativus*	*CsiT-1* (Csa017389)^CR^ *CsiT-2* (Csa017390)^CR^	Roots Leaves Stem Flower	Plasma membrane	Upregulated	Both mature and young leaves	[44]
*Cucurbita moschata Duch.*	*CmLsi1*(B+ ) (LOC111441250)	Roots (all root cells)	Plasma membrane	Upregulated	--	[36]
	*CmLsi1*(B-)	Shoot	Endoplasmic reticulum			
*O. sativa*	*OsLsi6* (Os06g0228200)	Root (All root cells) Shoot	Plasma membrane	Downregulated	Immature Root Tip	[34]
*H. vulgare*	*HvLsi6* (AB447484)	Root (Epidermis and cortex cells) Shoot Leaf blades and sheaths (Parenchyma cells and vascular bundle)	Plasma membrane	Upregulated	Root Tip (Vegetative growth stage) Nodes (Reproductive growth stage)	[37]
*Z. mays L*	*ZmLsi6* (AMQ98973)	Root (All root cells) Shoot (Xylem parenchyma cells) No polar localizationLeaf sheaths and blades (PL)	Plasma membrane	Unaffected		[14]
*Equistium arvense L.*	*EaLsi1* (NIP3;1)	Root	Plasma membrane			[95]
*Sorghum bicolor*	*SbLsi1*	Leaf blade (epidermal cells)	Plasma membrane			[74]
*Cannabis sativa*	CsaNIP2-1s *PKNIP2-1* (PK09456.1) *FNNIP2-1* (FN14156.1)	Aerial organs (flowers and shoot)	Plasma membrane			[57]
Efflux Silicon transporters						
*O. sativa*	*OsLsi2* (Os03g0107300)	Root Node (Bundle Sheeth Cells) PL	Plasma membrane	Upregulated		[28]
*H. vulgare*	*HvLsi2* (100502546)	Root Node (Parenchyma Cells)	Plasma membrane			[37]
*Z. mays L.*	*ZmLsi2* (541884)	Root	Plasma membrane			[14]
*C. moschata*	*CmLsi2-1* (AB551951) *CmLsi2-2* (AB551952)	Root Shoot	Plasma membrane		Young roots	[35]
*O. sativa*	*OsLsi3* (Os10g0547500)	Node (Parenchyma tissue)	Plasma membrane	Upregulated		[77]
*Chrysanthemum (Chrysanthemum X morifolium)*	*CmLsi2*					[98]
*E. arvense*	*EaLsi2-1*	RootShoot	Plasma membrane			[99]
*Glycine max*	*GmNIP2-1* (Glyma09G238200)	Root	Plasma membrane	Downregulated	--	[33]
*G. max*	*GmNIP2-2* (Glyma18G259500)	Shoot	Plasma membrane	Downregulated		
*C. sativus*	*CsaLsi21* (PK18630.1/FN08935.1) *CsaLsi2-2* (PK08860.1/FN07382.1) *CsaLsi2-3* (PK00413.1/FN13891.1)	Variable expression across plant tissues	Plasma membrane			[57]

CR = Circadian rhythm, NSCR = Not strongly controlled by Circadian rhythm, PL = Polar localization.
